# The MyD88+ Phenotype Is an Adverse Prognostic Factor in Epithelial Ovarian Cancer

**DOI:** 10.1371/journal.pone.0100816

**Published:** 2014-06-30

**Authors:** Charles J. d'Adhemar, Cathy D. Spillane, Michael F. Gallagher, Mark Bates, Katie M. Costello, Jacqui Barry-O'Crowley, Kathryn Haley, Niamh Kernan, Ciara Murphy, Paul C. Smyth, Ken O'Byrne, Stephen Pennington, Aoife A. Cooke, Brendan Ffrench, Cara M. Martin, Dearbhaile O'Donnell, Bryan Hennessy, Britta Stordal, Stephen Finn, Amanda McCann, Noreen Gleeson, Tom D'Arcy, Brian Flood, Luke A. J. O'Neill, Orla Sheils, Sharon O'Toole, John J. O'Leary

**Affiliations:** 1 Department of Histopathology, Trinity College Dublin, Dublin, Ireland; 2 Department of Pathology, Coombe Women's & Infants University Hospital, Dublin, Ireland; 3 Department of Obstetrics and Gynaecology, Trinity College Dublin, Dublin, Ireland; 4 Translational Research Institute, Princess Alexandra Hospital, Brisbane, Australia; 5 College of Health Sciences, University College Dublin, Belfield, Dublin, Ireland; 6 Department of Medical Oncology, St. James's Hospital, Dublin, Ireland; 7 Department of Medical Oncology, Royal College of Surgeons, Dublin, Ireland; 8 Department of Biochemistry, Trinity College Dublin, Ireland; University of Padova, Italy

## Abstract

The prognosis of epithelial ovarian cancer is poor in part due to the high frequency of chemoresistance. Recent evidence points to the Toll-like receptor-4 (TLR4), and particularly its adaptor protein MyD88, as one potential mediator of this resistance. This study aims to provide further evidence that MyD88 positive cancer cells are clinically significant, stem-like and reproducibly detectable for the purposes of prognostic stratification. Expression of TLR4 and MyD88 was assessed immunohistochemically in 198 paraffin-embedded ovarian tissues and in an embryonal carcinoma model of cancer stemness. In parallel, expression of TLR4 and MyD88 mRNA and regulatory microRNAs (miR-21 and miR-146a) was assessed, as well as in a series of chemosensitive and resistant cancer cells lines. Functional analysis of the pathway was assessed in chemoresistant SKOV-3 ovarian cancer cells. TLR4 and MyD88 expression can be reproducibly assessed via immunohistochemistry using a semi-quantitative scoring system. TLR4 expression was present in all ovarian epithelium (normal and neoplastic), whereas MyD88 was restricted to neoplastic cells, independent of tumour grade and associated with reduced progression-free and overall survival, in an immunohistological specific subset of serous carcinomas, p<0.05. MiR-21 and miR-146a expression was significantly increased in MyD88 negative cancers (p<0.05), indicating their participation in regulation. Significant alterations in MyD88 mRNA expression were observed between chemosensitive and chemoresistant cells and tissue. Knockdown of TLR4 in SKOV-3 ovarian cells recovered chemosensitivity. Knockdown of MyD88 alone did not. MyD88 expression was down-regulated in differentiated embryonal carcinoma (NTera2) cells, supporting the MyD88+ cancer stem cell hypothesis. Our findings demonstrate that expression of MyD88 is associated with significantly reduced patient survival and altered microRNA levels and suggest an intact/functioning TLR4/MyD88 pathway is required for acquisition of the chemoresistant phenotype. E*x vivo* manipulation of ovarian cancer stem cell (CSC) differentiation can decrease MyD88 expression, providing a potentially valuable CSC model for ovarian cancer.

## Introduction

Ovarian cancer is one of the most common and lethal cancers in women [1], [2], with incidences and mortality predicted to increase in western countries [3]. Epithelial ovarian cancers (EOCs) comprise the vast majority of adult ovarian malignancies, which are most commonly serous carcinomas [4], [5]. As ovarian cancer is often asymptomatic in its early stages or presents with vague symptoms mimicking extra-ovarian disease; most patients (70–75%) present with widespread disease at diagnosis with a resulting high mortality rate [6]. Current treatment options include surgery and platinum and/or taxane-based chemotherapy [7]. Although EOC typically responds very well to standard chemotherapy, with initial 70–80% response rates, this is frequently followed by recurrence that is often chemoresistant [8], [9]. Predicting and overcoming this chemoresistance remains a key challenge in treatment, however there are currently no available biomarkers (serum or tissue) that are truly predictive of behavior or chemoresponsiveness.

Toll-like receptors (TLRs) function as essential components of the innate immune system. They are membrane-bound receptors that recognize components of exogenous pathogens, such as bacterial lipopolysaccharide (LPS) and viral RNAs, leading to an inflammatory response. TLRs may also be activated by endogenous ligands including cellular debris derived from cancer progression [10]–[13]. Most TLRs signal via the myeloid differentiation primary response gene 88 (MyD88) and are expressed in both lymphoid and non-lymphoid tissues (predominantly in the former), with increasing evidence that they play important roles in cancer pathogenesis [14], [15]. The net effect of TLR signalling (+/−MyD88) is transcription factor activation, including nuclear factor-κB (NF-κB). NF-κB is a universally expressed transcription factor that is particularly important both as part of the normal inflammatory response and in tumourigenesis, regulating the expression of various inflammatory, apoptotic and oncogenic genes [16]. Ultimately NF-κB activation leads to increased production of cytokines, chemokines and growth factors. The activity of this pathway is normally kept in check, during the normal immune response, in part through microRNA regulation of TLR4 signaling, examples of which include microRNA 21 (miR-21) and miR-146a [17], [18], [19].

The TLR4/MyD88 pathway has in recent years been proposed as a risk factor for carcinogenesis and chemoresistance in ovarian cancer [20], [21]. While it has been observed that TLR4 expression is ubiquitous in EOC cells, a subgroup differentially expressing MyD88 has demonstrated increased cytokine/chemokine production and cellular proliferation upon activation of TLR4 [20]. Chen et al. [21] have used this differential expression to subdivide EOC into MyD88 positive and MyD88 negative. MyD88 positive EOCs have a functioning TLR4/MyD88 pathway and may represent an ovarian cancer stem cell (CSC) that is highly resistant to pro-apoptotic signaling and which can recruit leukocytes to actively promote a pro-inflammatory, pro-proliferative microenvironment [22]. MyD88 negative EOCs in contrast lack MyD88 and may represent more differentiated tumours that are less biologically aggressive [20], [23]. Alvero et al. [12] demonstrated that CSCs derived from ovarian tumour samples have a unique CD44+/MyD88+ phenotype, which facilitated resistance to both tumour necrosis factor (TNF) induced apoptosis and cytotoxic therapy. More recently MyD88 protein expression was shown to be a significantly poor prognostic factor in EOC [24].

The differential classification of MyD88 positive and negative EOC has implications for current therapeutic strategies. Although cisplatin-resistance occurs in a minority of women during their initial treatment (30%), it subsequently develops in all women receiving treatment for recurrent disease [9], and is most likely due to resistance to apoptotic signalling in MyD88 positive cells [12], [25], [26]. In addition, paclitaxel (a taxane-based agent) is a TLR4 ligand and can activate the TLR4/MyD88 pathway in MyD88 positive cells, actually inducing their proliferation [20], [27]. Such MyD88 competent cancer cells are theoretically able to exploit current chemotherapeutic strategies, by not only resisting their cytotoxic effects but also by using paclitaxel-ligation to facilitate their growth and that of the surrounding stroma via enhanced NF-κB production. Therefore current evidence suggests that paclitaxel may in fact be disadvantageous in patients whose tumours contain MyD88 positive EOC cells, despite retaining its cytotoxic effects in MyD88 negative cells [20], [27]. It is also possible that recurrent disease may represent a pool of more aggressive cancer cells (CSCs), which have effectively been ‘selected out’ by paclitaxel treatment.

We hypothesise that MyD88+ cancer cells represent a subpopulation of cells in EOC that confer more aggressive biological behaviour and chemoresistance. To date relatively small numbers of ovarian cancer samples, or isolated cancer cell lines, have been examined with respect to TLR4/MyD88 expression [20], [23], [27], [28]. The aim of this study was to comprehensively characterise the distribution and clinical significance of TLR4 and MyD88 expression in a cohort of ovarian neoplasms, which includes all common histologic subtypes and represents the largest such series studied to date [20], [23], [24], [27], [28]. The expression of two microRNAs known to regulate the TLR4/MyD88 pathway was also evaluated in a subset of malignant tumour samples to explore their role as epigenetic modulators of this pathway. Knockdown of TLR4/MyD88 was assessed to dissect the functional role of these genes. In parallel with the above experiments, TLR4/MyD88 expression was assessed in undifferentiated and differentiated embryonal carcinoma cells to provide further evidence of the cancer stem cell hypothesis in ovarian cancer.

## Results

### TLR4 and MyD88 Expression in the Test Series

**Figure 1:**
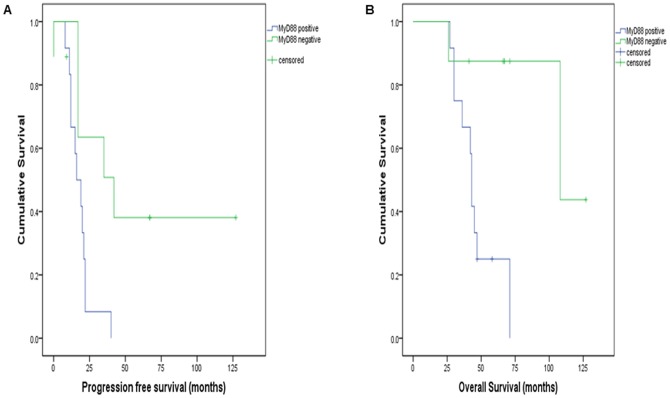
MyD88 expression and survival (Kaplan-Meier curves: median survival time shown in months). MyD88 positive tumours (n = 12) had significantly reduced progression-free survival (A) and overall survival (B) (p = 0.018 and p = 0.008, respectively).

The primary test series of malignant ovarian tumours comprised 20 patients with serous adenocarcinomas that were matched for grade (high-grade) and stage (FIGO III), and who had received platinum-based adjuvant chemotherapy. TLR4 expression was universally present and scored as positive (immunoscore >4) in all tumours (20/20). In contrast, positive immunostaining for MyD88 was observed in only 12/20 cases, and the remainder (8/20) were classified as MyD88 negative. Clinical follow-up data was available for all patients, 12 of whom had died at the time of this study, and retrospective analysis was performed to determine whether MyD88 expression was associated with patient survival. Progression free survival (PFS) and overall survival (OS) were assessed. Figure 1 demonstrates that MyD88 expression was associated with significantly shortened PFS and OS (p<0.05). The median time to recurrence (from the time of surgery) for patients with MyD88 positive tumours was 16 months, whereas MyD88 negative tumours recurred at a median time of 42 months (p = 0.018). MyD88 positive tumours were also associated with a markedly shorter OS. The median OS time for patients with MyD88 positive tumours was 43 months, compared to 108 months for MyD88 negative tumours (p = 0.008).

### TLR4 and MyD88 in the Validation Series

Expression of TLR4 and MyD88 was then assessed in 178 patient samples in order to validate the test series findings and to examine expression in non-malignant ovarian tissue and different histologic subtypes of EOC. These samples included normal ovaries (n = 50), benign cysts (n = 15), borderline tumours (n = 28) and malignant tumours (n = 85). Average patient age at time of surgery was 51 years for borderline tumours (range, 20-81) and 59 years for all malignant tumours (range, 23–94). The majority of malignant tumours were high-grade, with 62% assigned a grade of 3 (predominantly serous carcinomas), and high stage (51.3% FIGO stage IIIC, 8.1% stage IV). All tumours that were staged IIB and higher were serous carcinomas. The immunophenotype of each tissue type is summarized in Table 1.

**Table 1:** Distribution of TLR4 & MyD88 protein expression in all patient samples.

Ovarian Tissue	TLR Positive†	MyD88 Positive†
**NOSE (n = 50)**	7 (14%)	0
**Benign (n = 15)**	7 (47%)	3 (20%)
Serous (n = 5)	3 (60%)	3 (60%)
Mucinous (n = 5)	2 (40%)	0
Brenner (n = 5)	2 (40%)	0
**Borderline (n = 28)**	17 (61%)	12 (43%)
Serous (n = 15)	12 (80%)	12 (80%)
Mucinous (n = 13)	5 (38%)	0
**Malignant (n = 85)**	62 (73%)	40 (47%)
Serous (n = 69)	55 (80%)	40 (58%)
Mucinous (n = 6)	2 (33%)	0
Clear Cell (n = 5)	3 (60%)	0
Endometrioid (n = 5)	2 (40%)	0

Abbreviations: SD, standard deviation; FIGO, Federation International of Gynecology & Obstetrics; NOSE, normal ovarian surface epithelium.

† TLR4, MyD88 expression by immunohistochemistry (score >4 =  positive).

**Figure 2:**
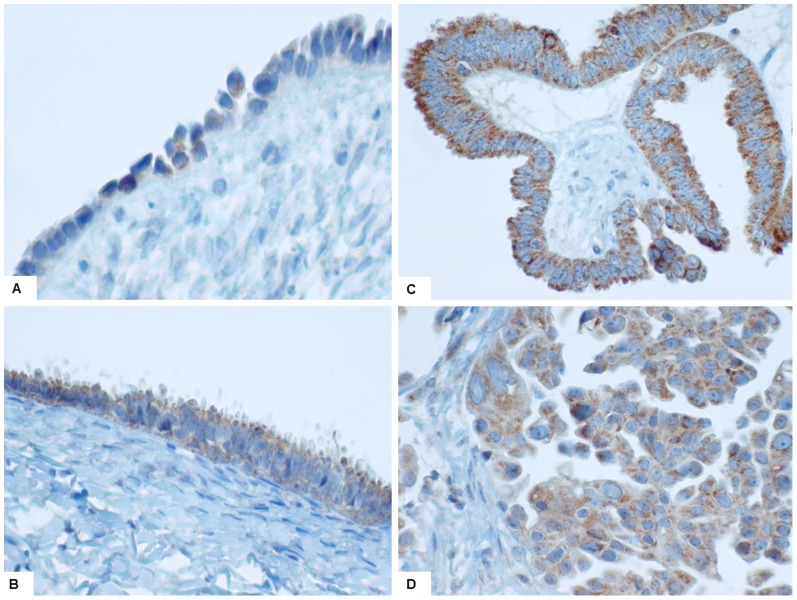
TLR4 expression pattern. A: focal weak staining in normal ovarian surface epithelium (40x); B: diffuse moderate staining in a benign serous cystadenoma (20x); C: diffuse strong staining in a borderline serous tumour (10x); D: diffuse strong staining in a (grade 2) serous carcinoma (40x).

**Figure 3:**
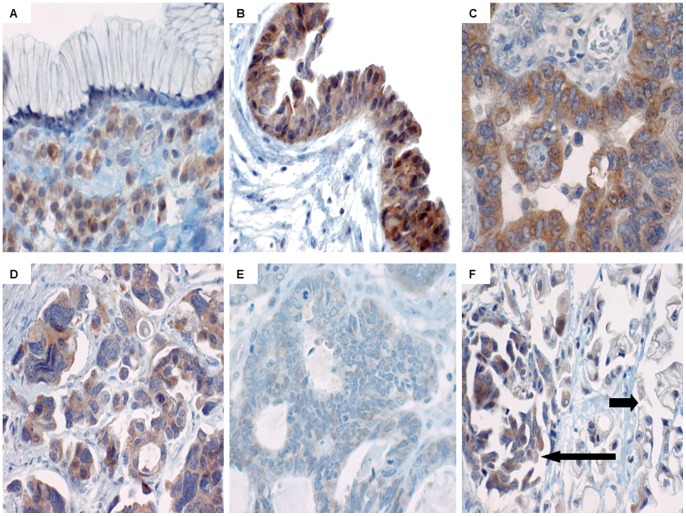
MyD88 expression pattern. A, negative benign mucinous cystadenoma, with positive stromal inflammatory cells (20x). B, positive borderline serous tumour (20x). C, positive low-grade serous carcinoma (40x). D, positive high-grade serous carcinoma (40x). E, negative endometrioid carcinoma (20x). F, positive serous carcinoma (long arrow) with component of negative clear cell carcinoma (short arrow) (40x).

The expression of both TLR4 and MyD88 was most uniform and strongest in serous neoplasms, particularly borderline and malignant serous tumours. Examples of TLR4 and MyD88 IHC expression are shown in Figures 2 & 3. Normal ovarian surface epithelium (NOSE) showed variable expression of TLR4 (7/50) but was uniformly negative for MyD88 (0/50). Approximately half of the benign cysts were TLR4 positive (7/15, mixed subtypes) but only a minority were MyD88 positive (3/15, all serous). The majority of borderline serous tumours were both TLR4 and MyD88 positive (12/15), with strong staining observed in 7/12. In contrast all borderline mucinous tumours were MyD88 negative. While 73% (62/85) of malignant tumours were TLR4 positive (mixed subtypes), co-expression of MyD88 was observed in only 47% cases, which were all serous carcinomas. All non-serous carcinomas (mucinous, clear cell and endometrioid) were MyD88 negative. This data generated 40 MyD88 positive and 45 MyD88 negative cancers. MyD88 expression was observed in both poorly- and well-differentiated tumour cells, and was strong near areas of necrosis. As shown in Table 2 there was no correlation between patient age or overall grade of differentiation and TLR4 or MyD88 expression. Collectively, the TLR4+/MyD88- phenotype was associated with all types of tissue, including non-dysplastic epithelium, while TLR4+/MyD88+ (co-expression) was associated only with ovarian neoplasms.

**Table 2:** Characterisation of TLR4 & MyD88 expression in ovarian cancer.

Malignant Tumours	TLR4 Positive†	TLR4 Negative†	MyD88 Positive†	MyD88 Negative†
**Patients (n = 85)**	62	23	40	45
**Mean age (y) ±SD**	60±13	57±15	61±12	56±15
**FIGO stage** ‡				
I	8 (11%)	10 (13%)	3 (4%)	13 (18%)
II	2 (3%)	3 (4%)	2 (3%)	3 (4%)
III	25 (33%)	21 (28%)	27 (38%)	19 (26%)
IV	4 (5%)	2 (3%)	5 (7%)	1 (1%)
**Grade of Differentiation**				
1	1 (33%)*	2 (67%)	0	3 (100%)
2	13 (65%)	7 (35%)	12 (60%)	8 (40%)
3	17 (45%)	21 (55%)	18 (47%)	20 (53%)

Abbreviations: SD, standard deviation; FIGO, Federation International of Gynecology & Obstetrics.

† TLR4, MyD88 expression by immunohistochemistry (score >4 =  positive).

‡ Simplified FIGO staging.

*% of each grade.

**Figure 4:**
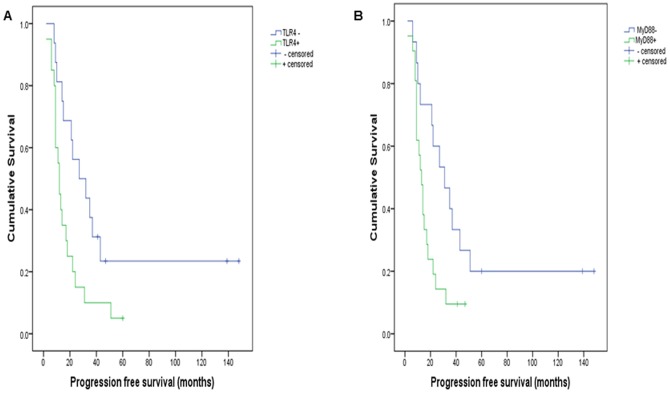
TLR4/MyD88 and progression-free survival (Kaplan-Meier curves; median survival shown in months). TLR4 (A) or MyD88 (B) negative cases had significantly better PFS (15 & 18 months longer; p<0.05).

**Figure 5:**
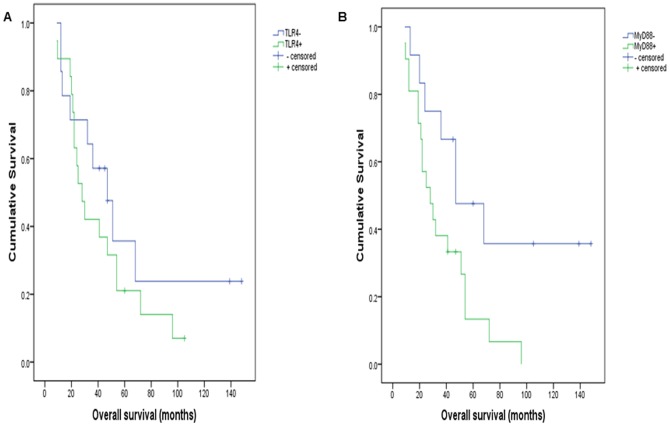
TLR4/MyD88 and overall survival (Kaplan-Meier curves; median survival shown in months). Survival was longer in MyD88 (B) negative cases (by 19 months; p<0.05). The difference in survival associated with TLR4 (A) is not significant (p>0.5).

Retrospective analysis was performed to determine whether TLR4/MyD88 expression influenced patient survival in this larger series. Clinical follow-up data was available for 39 patients with malignant tumours, 33 of whom had died at the time of this study. Cases excluded from follow-up included non-serous carcinomas (all MyD88 negative), those with recurrent disease (n = 2), patients who died of post-operative complications or unrelated causes (n = 2) and patients lost to follow-up (further treatment and follow-up at a different institution). Selected cases were classified into 2 groups based on MyD88 protein expression: Group 0 (MyD88 negative); Group 1 (MyD88 positive). All cases were grade and stage-matched (grade 3, FIGO III/IV), were optimally debulked and patients had received adjuvant platinum-based chemotherapy with (n = 28) or without (n = 11) paclitaxel. As illustrated in Figures 4 & 5, TLR4 and MyD88 expression were both associated with significantly shortened progression-free survival (PFS), and MyD88 expression was associated with reduced overall survival (p<0.05). The median time to recurrence for patients with tumours that expressed TLR4 was 12 months (n = 20), whereas TLR4 negative tumours recurred in a median time of 27 months (n = 19); p = 0.016. Similarly, median time to recurrence for MyD88 positive tumours was 13 months (n = 21), compared to 31 months if MyD88 negative (n = 18); p = 0.02. The difference in OS between TLR4 positive (n = 19) and TLR4 negative tumours (n = 14) was not statistically significant (p = 0.312). The median OS time for patients with MyD88 positive EOC was 28 months (n = 21), while patients with MyD88 negative tumours (n = 12) had significantly longer overall survival (47 months, p = 0.029). MyD88 positive malignancies therefore were associated with reduced patient survival and disease-free intervals.

### MicroRNA Expression in the Test Series

**Figure 6:**
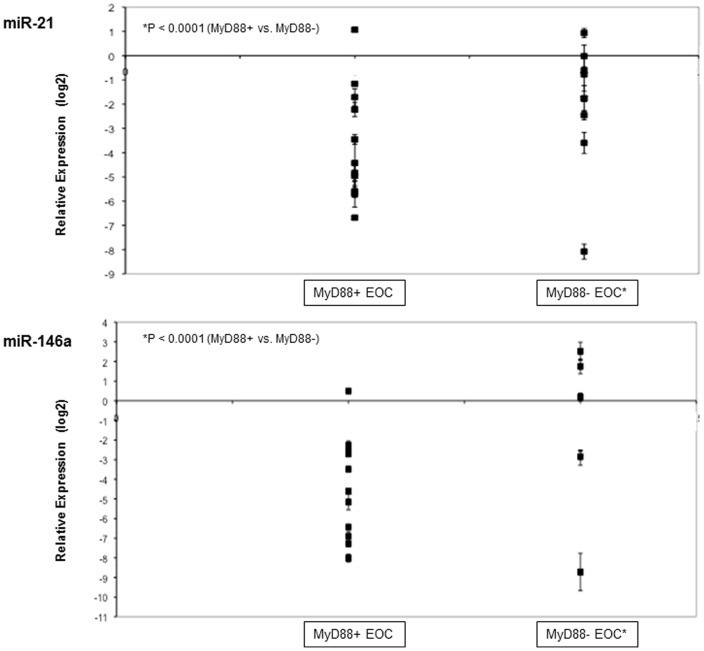
MiR-21 & miR-146a expression in the test series. Scatter plots showing relative microRNA expression with standard deviation (fold changes calculated via the 2^−ΔΔCt^ method). 20 EOC cases (serous carcinomas) grouped as MyD88+ or MyD88- based on protein expression; data shown relative to each group. Average expression of miR-21 & miR-146a increased in MyD88 negative EOC (p<0.05).

EOC samples from the initial test series were divided into two groups based on previous immunohistochemical results (MyD88 positive, n = 11; MyD88 negative, n = 9). As shown in Figure 6, the average expression of miR-21 and miR-146a in MyD88 negative cancers was increased relative to the average of the MyD88 positive group (p<0.001).

### Gene and microRNA Expression in the Validation Series

**Figure 7:**
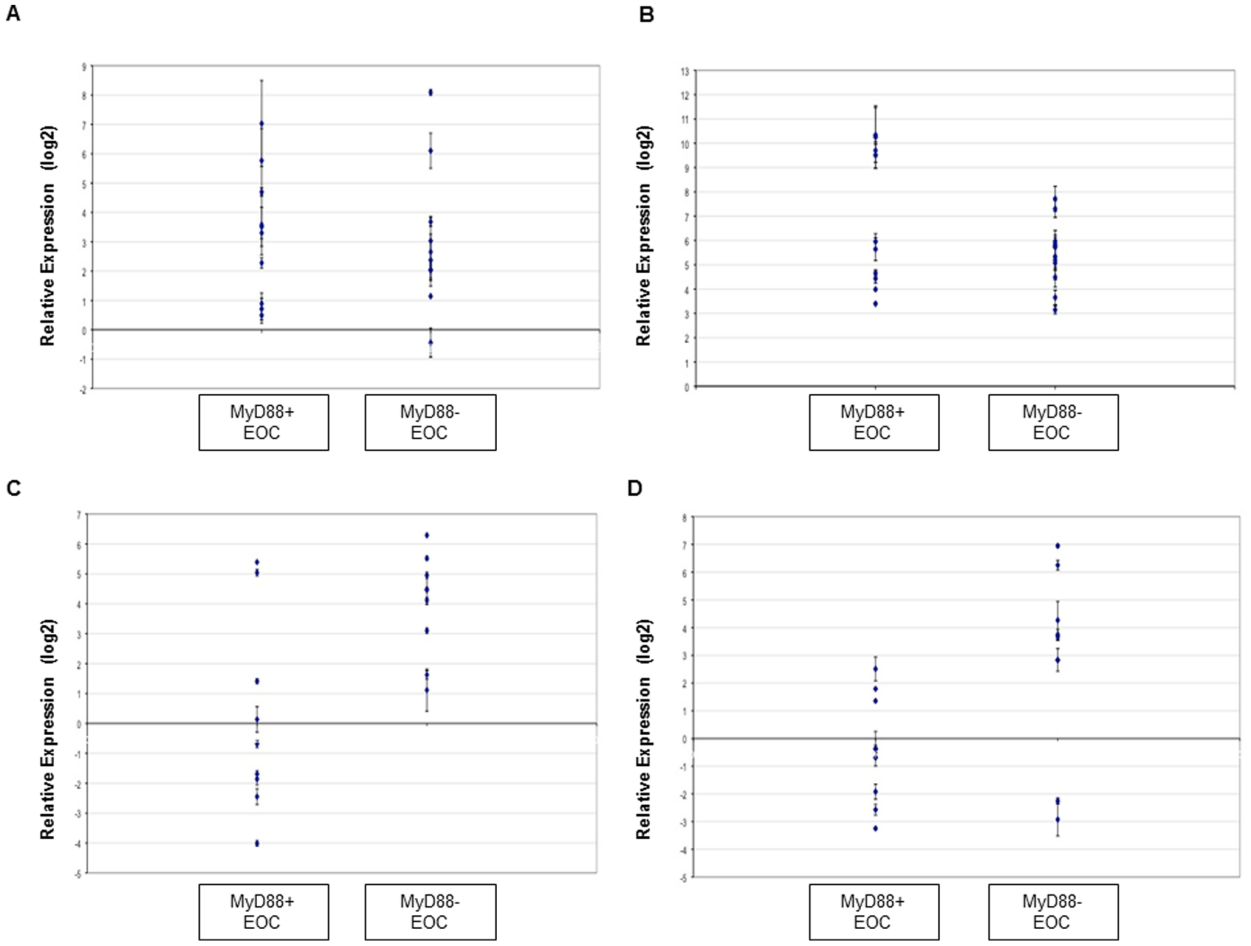
TLR4/MyD88 mRNA and miR-21 & miR-146a expression. Scatter plots showing relative gene and microRNA expression with standard deviation (fold changes calculated via the 2^−ΔΔCt^ method). 22 EOC cases (serous carcinomas) grouped as MyD88+ or MyD88- based on protein expression; data shown relative to each group. A & B, TLR4 & MyD88 mRNA statistically unchanged between MyD88 positive & MyD88 negative groups (p>0.05); C & D, levels of both miR-21 & miR-146a up-regulated in MyD88 negative EOC (p<0.05).

Selected malignant patient tumour samples were divided into two groups based on previous IHC results (MyD88 positive, n = 10; MyD88 negative, n = 12). Expression of miR-21 and miR-146a was analyzed in parallel with TLR4 and MyD88 gene expression analysis. Figure 7 shows that, similar to the test series, expression of both microRNAs was significantly increased in MyD88 negative cancers relative to the MyD88 positive group (miR-21, p = 0.0046; miR-146a, p = 0.0191), with more biological heterogeneity observed with miR-146a levels. The associated levels of TLR4 & MyD88 mRNA were statistically unaltered between the MyD88 positive and negative cancers (p = 0.8777 and 0.1348, respectively). Therefore miR-21 and miR-146a appear to be inversely linked to MyD88 in ovarian cancer. The absence of MyD88 mRNA alteration in the MyD88 negative group suggests that both miR-21 and miR-146a are targeting MyD88 at the post-transcriptional level.

### TLR4 and MyD88 expression in a cohort of responders and non-responders

MyD88 mRNA was upregulated 4.9 fold in a cohort of non-responders compared to responders (p<0.05). Similarly, TLR4 mRNA was upregulated 3.4 fold in this cohort (p<0.05). All patients analysed here had advanced serous papillary adenocarcinomas and received carboplatin and paclitaxel as first line chemotherapy following optimal debulking surgery.

### Gene and miRNA expression in cell lines

**Figure 8:**
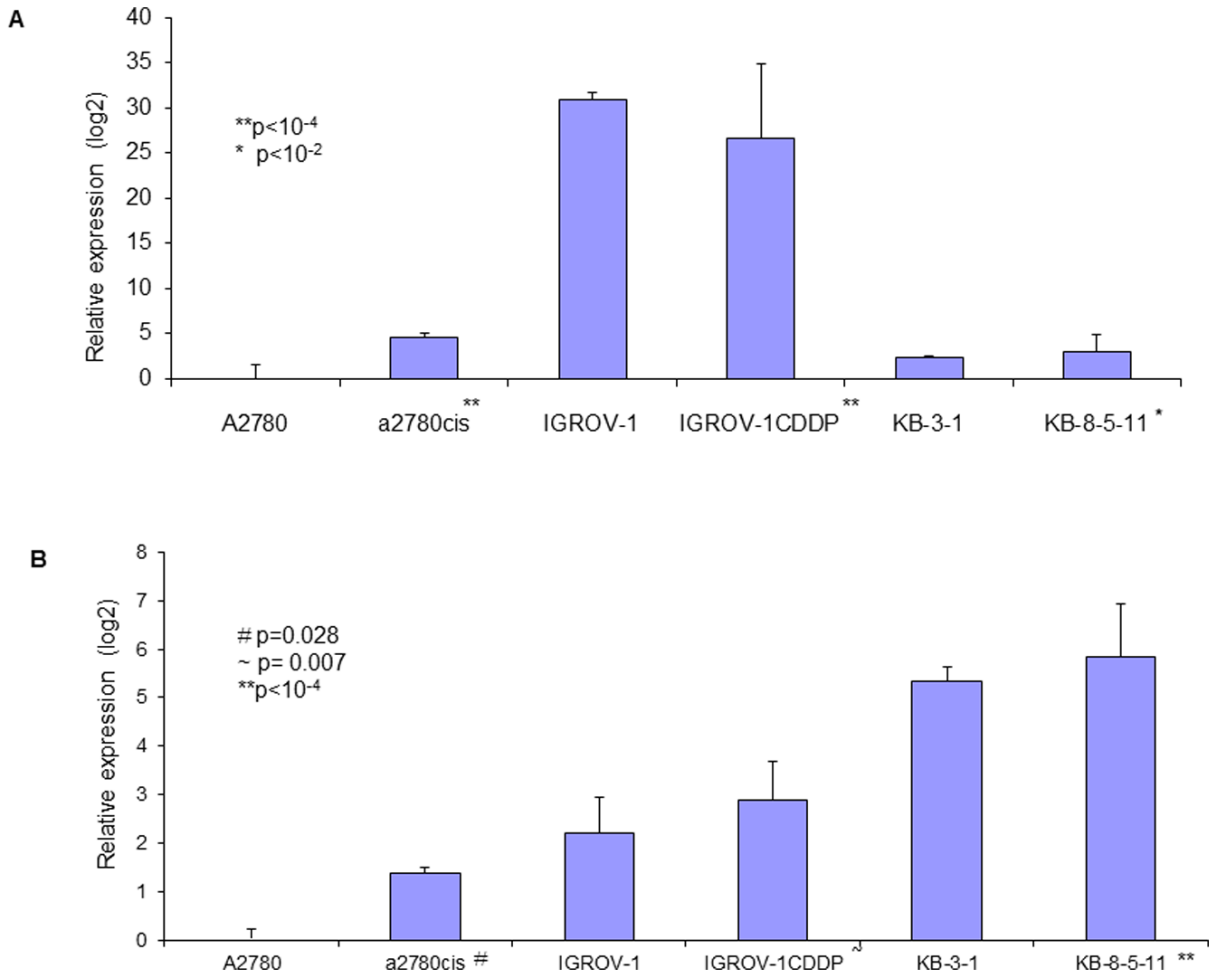
Changes in TLR4 (A) and MyD88 (B) mRNA expression between chemosensitive and chemoresistant cancer cells. Data are expressed as fold change in expression with respect to A2780 cancer cells (with standard deviation).

Significant changes in TLR4 mRNA expression were observed between chemosensitive and chemoresistant cells, although the degree of change was variable (Figure 8A). A2780cis cells showed a 24.1 fold increase in TLR4 compared to A2780. In contrast there was an 18.8 fold decrease in TLR4 in the chemoresistant IGROV-1CDDP cells relative to IGROV-1. KB-8-5-11 cells showed the smallest change from their parent line with only a 1.6 fold increase in TLR4.

Increased MyD88 mRNA expression was observed in all chemoresistant cancer cell lines; these changes were small but statistically significant (Figure 8B). There was a 2.6 fold increase in MyD88 in A2780cis cells compared to MyD88-negative A2780 cells. IGROV-1CDDP cells showed a 1.6 fold increase in MyD88 relative to the chemosensitive IGROV-1 cells. Similar to the TLR4 data, the change in MyD88 expression in KB-8-5-11 cells from the chemosensitive KB-3-1 parent line was the smallest with only a 1.4 fold increase in MyD88.

**Figure 9:**
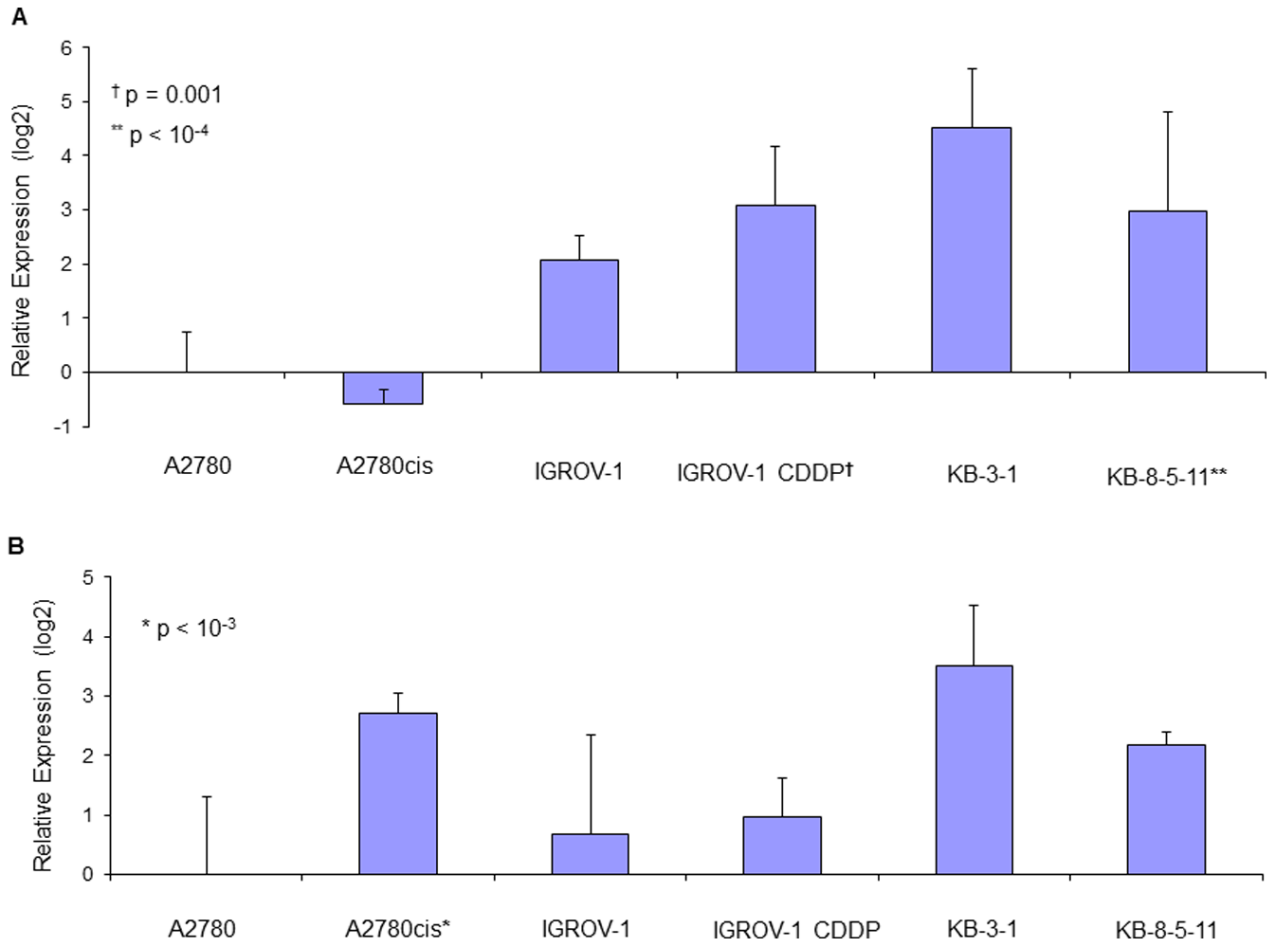
miR-21 (A) and miR-146a (B) expression in chemosensitive and chemoresistant cancer cells. Data are expressed as fold change in expression with respect to A2780 cancer cells (with standard deviation).

Variable changes in the levels of miR-21 were observed between chemosensitive and chemoresistant cells (Figure 9A). A2780cis cells showed a 1.5 fold decrease in miR-21 compared to A2780, however this change was not statistically significant (p = 0.5958). A 2 fold increase in miR-21 was detected in the chemoresistant IGROV-1CDDP cells relative to IGROV-1. KB-8-5-11 cells showed a 2.9 fold decrease in miR-21 compared to their parent (chemosensitive) line.

Similar to miR-21, changes in miR-146a levels between chemosensitive and chemoresistant cancer cells were variable (Figure 9B). There was a 6.5 fold increase in miR-146a in A2780cis cells compared to MyD88-negative A2780 cells. IGROV-1CDDP cells showed a 1.2 fold increase in MyD88 relative to the chemosensitive IGROV-1 cells, however this change was not statistically significant (p = 0.4557). A 2.5 fold reduction in miR-146a was detected in KB-8-5-11 cells compared to their chemosensitive (KB-3-1) parent line.

### Reduced TLR4 expression results in increased chemosensitivity of ovarian cancer cells

**Figure 10:**
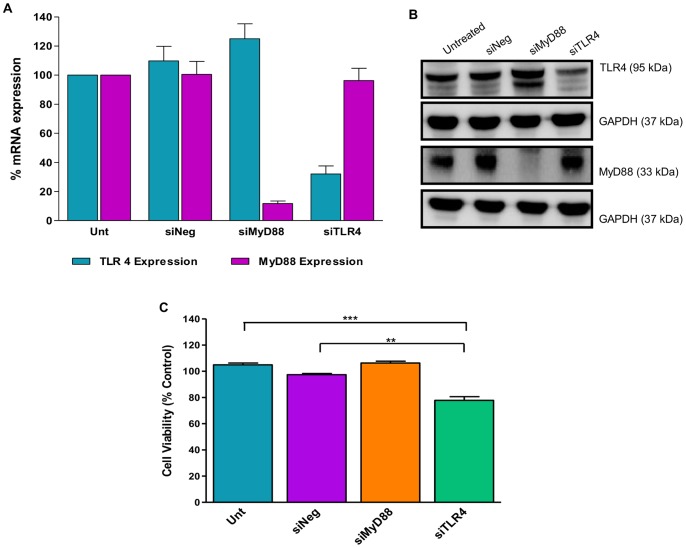
The effect of silencing MyD88 and TLR4 mRNA on the chemoresponsive properties of SKOV-3 cells. SKOV-3 cells were left untransfected (Unt), transfected with negative control siRNA (siNeg), MyD88 targeting siRNA (siMyD88) or TLR4 targeting siRNA (siTLR4). The transfected cells were incubated for 72 hrs before either harvesting for mRNA analysis (A), for protein analysis (B) or treatment with paclitaxel (C). (A) MyD88 and TLR4 mRNA expression levels were evaluated by TaqMan RT-PCR. MyD88 and TLR4 mRNA expression was normalised to that of an endogenous control, B2M, and calibrated to that of untreated cells to establish the relative percentage of mRNA expression (n = 3, mean +SD). (B) MyD88 and TLR4 mRNA expression levels were evaluated by western blot analysis. GAPDH was used as a loading control. (C) Transfected cells were either left untreated, treated with DMSO (vehicle control) or 3.5 nM of paclitaxel (IC25). 48 hrs post treatment, cell viability was assessed by means of the CCK-8 assay. % cell viability rate was calculated by comparing the absorbance values for the vehicle control to the corresponding paclitaxel treated samples. Results are expressed as mean +SD, n = 3; *p<0.05, **p<0.01 (un-paired Student's t-test).

To functionally evaluate the role of the TLR4/MyD88 pathway on the chemosensitivity of ovarian cancer cells the chemoresistant ovarian cancer cell line SKOV-3 was transfected with siRNA specifically targeting MyD88 or TLR4. After 72 hrs MyD88 was significant decreased at both the mRNA (Figure 10A) and protein (Figure 10B) level in the cells transfected with siMyD88. However, the loss of MyD88 expression had no effect on the chemosensitivity of the cells (Figure 10C). Significant reductions in the expression of TLR4 at the mRNA (Figure 10A) and protein (Figure 10B) level was also observed in cells 72 hrs after transfection. However, the reduced level of TLR4 expression significantly affected the response of the SKOV-3 towards paclitaxel (Figure 10C). There was a 27.1% decrease in cell viability in siTLR4 transfected paclitaxel treated cells compared to untransfected paclitaxel treated cells and a 19.6% decrease when compared to siNeg transfected cells. Thus, the silencing of TLR4 generated a more chemosensitive phenotype in the SKOV-3 ovarian cancer cells.

### Embryonal carcinoma cells show reduced MyD88 expression following differentiation

**Figure 11:**
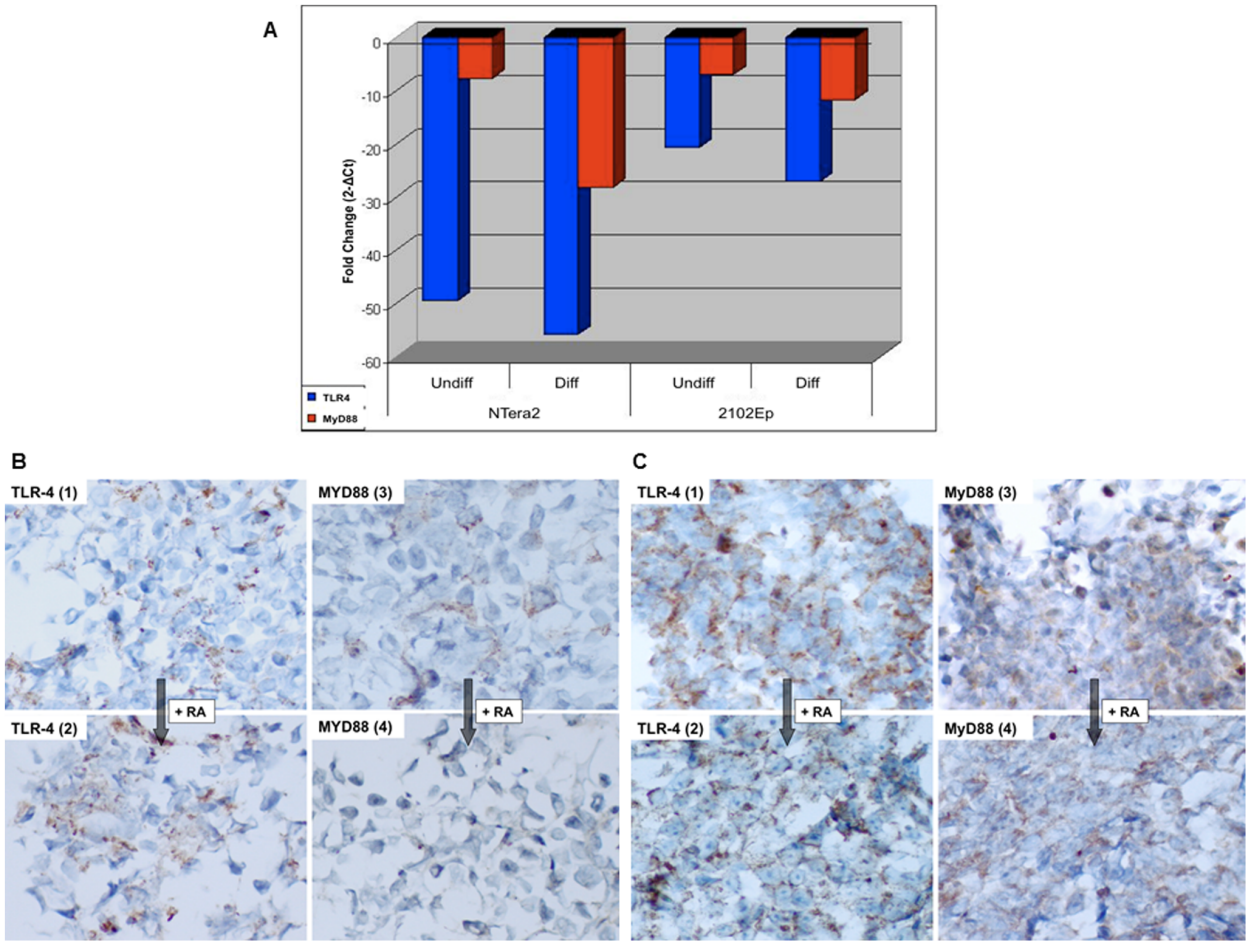
TLR4 and MyD88 expression in undifferentiated and retinoic acid (RA)-differentiated NTera2 and 2102Ep cells. **A**, qPCR data: MyD88 expression is down-regulated upon NTera2 differentiation, with minimal changes in TLR4; in contrast 2102Ep cells avoid differentiation and maintain both TLR4 & MyD88 expression (fold changes shown are proportional to internal control gene GAPDH and calculated via the 2^−ΔΔCt^ method). **B**, TLR4 and MyD88 protein expression in NTera2 cells: TLR4 staining was unchanged following differentiation (left panel); MyD88 staining in undifferentiated NTera2 cells showed significantly reduced staining after differentiation (right panel) (all 40x). **C**, TLR4 and MyD88 expression in 2102Ep cells: minimal changes in TLR4 (left panel) or MyD88 (right panel) were observed following RA-treatment (all at 40x).

Data from a large-scale microarray experiment (M Gallagher, unpublished data) involving embryonal carcinoma cancer stem cells (NTera2 and 2102Ep) was interrogated for differential TLR4-signalling gene expression, which indicated that MyD88 expression was high in undifferentiated NTera2 cells and decreased rapidly upon differentiation in retinoic acid (RA). To validate microarray data, undifferentiated and differentiated NTera2 and 2102Ep samples were assessed for MyD88 gene and protein expression. As TLR4 was hypothesised to regulate MyD88 in these cells, the expression of TLR4 was also assessed despite constitutive expression in the array studies. Down-regulation of MyD88 upon differentiation of pluripotent NTera2 cells was confirmed across multiple time courses (Figure 11A). TLR4 was constitutively expressed, validating array data. In contrast, the expression of TLR4 and MyD88 was unaltered in nullipotent 2102Ep cells treated with RA (Figure 11A). These alterations were mirrored at protein level (Figure 11B,C). MyD88 protein expression was significantly reduced following differentiation of NTera2 cells (MyD88-), whereas no significant change was observed in TLR4 staining (Figure 11B). In contrast 2102Ep cells, both undifferentiated and treated with retinoic acid, were TLR4+, MyD88+ (Figure 11C). In terms of associated microRNA alterations, our group has previously demonstrated that while miR-21 is unaltered in either cell line in response to RA, miR-146a is up-regulated during differentiation of NTera2 cells but unchanged in 2102Ep cells [29]. This suggests that, similar to EOC, miR-146a is inversely linked to MyD88 in differentiated embryonal carcinoma cancer stem cells.

## Discussion

The characterisation here of TLR4 and MyD88 expression in epithelial ovarian neoplasia has shown that while TLR4 expression of some degree is ubiquitous to all types of ovarian epithelium, including non-dysplastic epithelium, MyD88 is only expressed in neoplastic tissue. Such a restriction of MyD88 to neoplastic ovarian tissue corroborates similar findings recently reported in the literature [24]. However in this study, TLR4/MyD88 co-expression showed a predilection for serous neoplasms in general, particularly borderline and malignant serous tumours, and was expressed in both high- and low-grade serous carcinomas. In contrast TLR4 in the absence of MyD88 was associated with non-malignant tissue, non-serous benign and borderline tumours, a subgroup of serous carcinomas and non-serous carcinomas (MyD88 negative EOC). As TLR4 expression was present in all types of ovarian epithelium, it appears that TLR4 signalling is linked to either malignant serous tumours or non-serous tumours proportional to being MyD88-dependent or -independent.

Chen et al. [21] originally postulated that the ratio of MyD88 positive and negative EOC cells may determine tumour characteristics in terms of progression, chemoresistance and recurrence. This is entirely in keeping with our results illustrating the distribution of MyD88 expression in ovarian neoplasia and associations with patient survivial. MyD88 positive cancers analysed in this study recurred 18 months earlier than MyD88 negative cancers, with a similar reduction in overall survival of 19 months (p<0.05). The results of this analysis are in exact concordance with recent reports indicating reductions in disease-free intervals and patient survival with MyD88 positive cancers, ranging from 11–35 month reductions in progression-free survival [20], [24], [27], [28] and 26–36 month reductions in overall survival [24], [27]. The very large difference in survival that was observed in the test series relative to MyD88 expression is likely due to the small sample size in this cohort, as it is markedly discordant with all previous studies and the validation series examined here.

Furthermore the changes observed in MyD88 expression in embryonal carcinoma (EC) cancer stem cells, one of the best characterized CSC models in use today [30]–[32], also suggest that MyD88 positive cancer cells are more biologically aggressive than MyD88 negative cells. Undifferentiated stem cell-like populations from malignancies are often sufficient to efficiently regenerate tumours. NTera2 EC cells differentiate *in vivo* to form teratocarcinomas, three germ layer tumours that can arise in the ovary [33]. NTera2 cells are highly-tumourigenic in the undifferentiated state, a property that is greatly reduced or eliminated upon differentiation [32], [33]. In contrast, 2102Ep EC cells can avoid differentiation *in vivo* to generate highly aggressive pure embryonal carcinomas [31]. We have shown that while TLR4 is constitutively expressed in EC cells, loss of MyD88 expression is associated with a ‘differentiation switch’: transition of NTera2 EC cells from their highly-tumourigenic (undifferentiated) to less-tumourigenic (differentiated) states. This change in expression upon differentiation is subtle but highly reproducible, and demonstrates that *ex vivo* manipulation of ovarian CSC differentiation can decrease MyD88 expression. We believe that it supports the hypothesis that EOC MyD88 positive cells may be stem-like due to their MyD88 expression pattern [12], [21] and also demonstrates a practical and easily manipulated cancer stem model with relevance to ovarian cancer.

It has recently been reported that miR-21 regulates TLR4 by targeting the tumour suppressor protein PDCD4 [19], whereas miR-146a is NF-κB-dependent [34] and targets and reduces the expression of IRAK-1, IRAK-2 and TRAF-6, which are key MyD88 pathway components [18], [35]. Ultimately miR-21 and miR-146a mutually participate in the regulation of TLR4/MyD88 expression, serving as negative regulators of MyD88-dependent TLR4 signalling. The novel analysis of miR-21 and miR-146a expression in this study, based on the categorization of EOC into MyD88 positive and MyD88 negative, demonstrates that expression of both MyD88 and these microRNAs are linked to ovarian cancer. Subtle but significant alterations in MyD88 mRNA expression were observed between chemosensitive and chemoresistant ovarian and cervical cancer cells. These findings demonstrate that MyD88 expression is linked to miR-21 and miR-146a, and provides further support to previous observations that MyD88 is associated with adverse biological characteristics including resistance to standard platinum and taxane- based chemotherapies, with consequent reduced survival. Examining macro-dissected FFPE sections from responders and non-responders demonstrated significant increases in TLR4 and MyD88 mRNA in non-responders. Knockdown of TLR4 in SKOV-3 ovarian cells recovered chemosensitivity to paclitaxel. Knockdown of MyD88 alone did not. The results suggest that an intact/functioning TLR4/MyD88 pathway is required for the acquisition of the chemoresistant phenotype.

In summary, this data provides additional support to the hypothesis that MyD88 expression is an adverse prognostic factor in ovarian cancer, for which evidence has been mounting in recent studies. Similar to Zhu et al. [24], we have demonstrated a reproducible method of assessing MyD88 status by immunohistochemistry, lending weight to its use as a practical prognostic marker in EOC and which should assist future prospective studies. In addition, MyD88 staining has being optimised for cytological analysis of ascites which could have an important role to play in ovarian cancer treatment, in particular in the neoadjuvant setting. Specific associations with tumour subtype, stage, patient survival, microRNA regulation and cancer stem cell differentiation have been demonstrated.

## Materials and Methods

### Ethics Statement

Informed consent was obtained from patients. The study was approved the St. James's Hospital/Adelaide and Meath Hospital Dublin, incorporating the National Children's Hospital Research Ethics Committee.

### Patient and sample selection

Samples of ovarian tissue from 198 patients who underwent surgery from 1993–2008, in the form of formalin-fixed paraffin-embedded (FFPE) tissue blocks, were accessioned from the archives of St. James's Hospital (SJH), Dublin, Ireland and the Adelaide and Meath Hospital incorporating the National Children's Hospital (AMNCH), Dublin. A test set of 20 malignant tumours (serous adenocarcinomas) was initially selected for TLR4 and MyD88 expression analysis. The subsequent validation series included 50 normal ovaries and 128 neoplasms. The latter include benign serous cystadenomas (n = 5); benign mucinous cystadenomas (n = 5); benign Brenner tumours (n = 5); borderline serous tumours (n = 15); borderline mucinous tumours (n = 13); serous adenocarcinomas (n = 69); mucinous adenocarcinomas (n = 6); clear cell adenocarcinomas (n = 5); endometrioid adenocarcinomas (n = 5). Clinicopathological information was obtained via a retrospective chart review and access to the local laboratory information systems.

### Immunohistochemistry

TLR4 and MyD88 antibodies were purchased from Santa Cruz Biotechnology, Inc. (Santa Cruz, CA, USA) at initial concentrations of 200 µg/mL: rabbit polyclonal antibody (RpAb) to TLR4 (clone H-80); RpAb to MyD88 (clone HFL-296). 6 µm sections were cut from each FFPE block and the expression of TLR4 and MyD88 was assessed using the Ventana Benchmark LT DAB system (Ventana Medical Systems, AZ, USA). Standardised protocols were established for each antibody using a high-grade breast (ductal) carcinoma as a positive control, as per the manufacturer's recommendations. Antibody concentrations, initially at 200 µg/mL, were both at 1∶30 dilution. Briefly, each protocol included 60-minute standard cell conditioning using CC1 antigen retrieval buffer (Ventana), followed by manual antibody titration and incubation for 32 minutes at 37°C.

### Assessment of Immunohistochemical Staining

**Figure 12:**
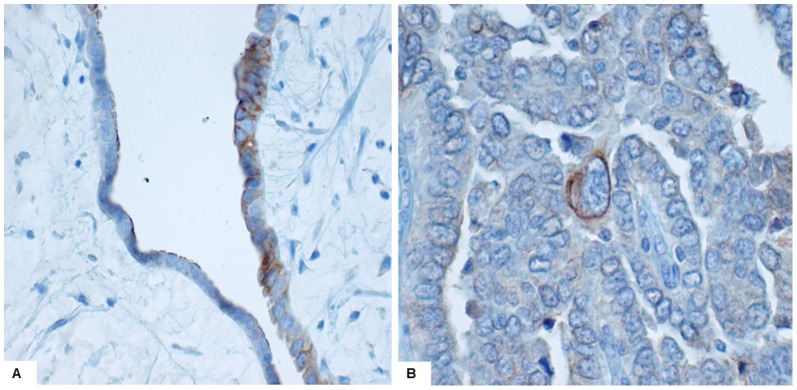
Heterogeneous expression of monoclonal anti-TLR4. A: variable staining observed in adjacent epithelium within a benign serous cystadenoma (20x). B: focal strong staining within a serous carcinoma (40x).

**Figure 13:**
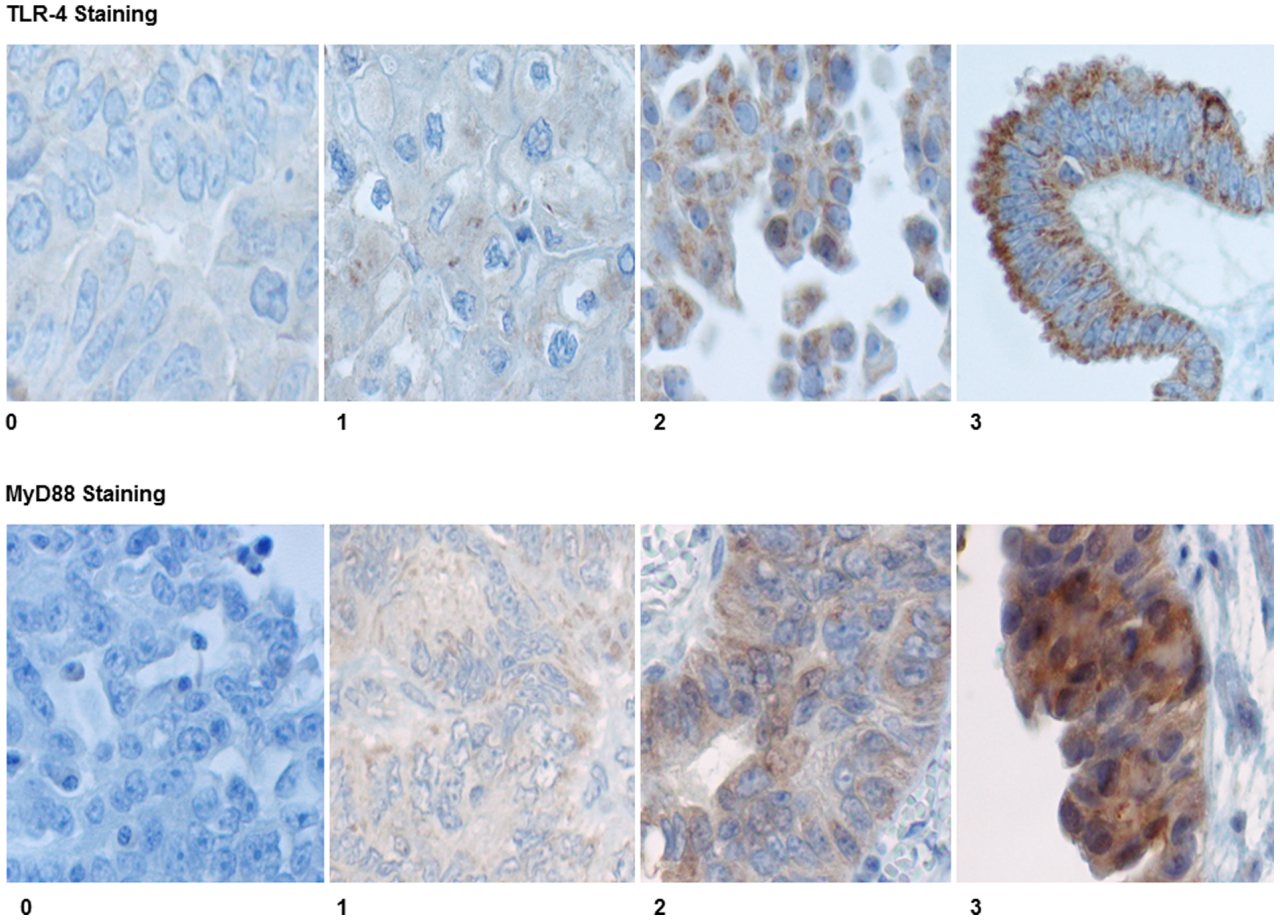
Quantification of immunohistochemical staining of TLR4 and MyD88 (0 =  no staining, 1 =  weak staining, 2 =  moderate staining, 3 =  strong staining).

Initial immunohistochemistry staining for TLR4 (cytoplasmic and membranous) and MyD88 (cytoplasmic) was highly variable, with marked heterogeneity noted within the same sample. This was not geographical but showed distinct and repeated variation between adjacent tumour cells (Figure 12). In an effort to account for this heterogeneity, a novel visual semi-quantification method was employed. This method was developed in order to accurately quantify the expression of TLR4 and MyD88 even in cases with heterogeneous staining, and was derived from similar methods employed in other studies [24], [36], [37], [38]. In addition whole paraffin sections were examined as focal protein expression could potentially be missed in tissue microarrays. Staining was based on immunointensity (II) and immunopositivity (IP). Immunointensity was scored as: 0, negative; 1, weak; 2, moderate; 3, strong (Figure 13). The percentage of immunopositive cells was scored as: 1 (1–10%); 2 (11–40%); 3 (41–70%); 4 (>70%). The product of II and IP produced values ranging from 0 to 12; a cut-off value of >4 determined IHC positivity. Stained slides were assigned random numbers and graded blind by two pathologists.

### RNA and miRNA Extraction and Qualitative PCR (qPCR) from FFPE samples

**Figure 14:**
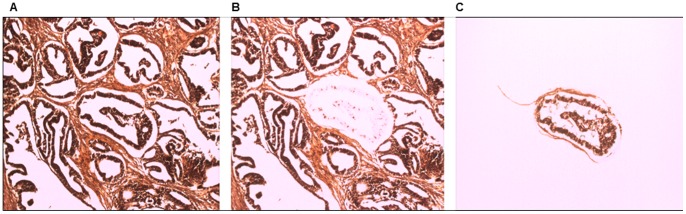
Photomicrographs illustrating the process of laser capture microdissection , including pre-dissection (A), post-dissection with a malignant gland removed from the surrounding stroma (B) and the isolated epithelial sample for genetic analysis (C).

A subset of malignant patient samples (n = 32) was selected for gene and miRNA expression analysis. Tumour cells were isolated from the surrounding stroma using laser capture microdissection (LCM). In brief, 7 µm sections were cut from each FFPE block and following haematoxylin and eosin (H&E) staining for visualisation, tumour epithelial cells were selected and extracted using LCM (Arcturus XT Microdissection Instrument, MDS Analytical Technologies, CA, USA) (Figure 14). cDNA was prepared with the High-Capacity cDNA Archive and TaqMan MicroRNA RT kits according to manufacturers' instructions (Applied Biosystems, CA, USA). For analysis of gene expression individual mRNAs were monitored with the following inventoried TaqMan assays (Applied Biosystems): human GAPDH assay (Hs99999905_m1), human TLR4 assay (Hs00152939_m1) and human MyD88 assay (Hs00182082_m1). For miRNA analysis, individual miRNA TaqMan assays for the endogenous reference RNA RNU6B, miR-21 and miR-146A were done according to the manufacturer's instructions (Applied Biosystems). Amplification and analysis were performed on the ABI Prism 7500 Fast Sequence Detection Systems (Applied Biosystems). All assays were done in triplicate. Changes in expression were calculated by the change in threshold (ΔΔCt) method [39] with GAPDH as the endogenous control for gene expression analysis and RNU6B as the endogenous control for miRNA analysis.

In addition, a cohort of 27 macro-dissected advanced serous cancers were interrogated for TLR4 and MyD88 mRNA expression as described above. All cases were optimally debulked and expression was measured relative to their chemotherapy response status. Chemoresponsive or ‘Responders’ were defined as patients who had a disease free interval since completion of combination chemotherapy for 12 months or longer. Chemoresistant patients, or ‘Non-Responders’ were defined as patients who experienced a relapse of disease within six months of completing their chemotherapy regimen.

### RNA and miRNA Extraction and Qualitative PCR (qPCR) from cell lines

TLR4 and MyD88 gene expression was assessed in epithelial ovarian cancer cells A2780 and IGROV-1, and cervical cancer cells KB-3-1, as well as their chemoresistant daughter cells A2780cis (cisplatin-resistant), IGROV-1CDDP [40], [41] (cisplatin & paclitaxel-resistant) and KB-8-5-11 [42] (colchicine and paclitaxel-resistant). IGROV-1 and IGROVCDDP cells were grown in antibiotic and chemotherapy-free RPMI (Sigma #R8758) with 10% FCS (Lonza, Belgium). KB-3-1 and its colchicine-resistant variant KB-8-5-11 were grown in DMEM (Sigma #D5671), containing 1% penicillin-streptomycin, 2% L-glutamine and 1% sodium pyruvate with 10% FCS (Lonza). KB-8-5-11 cells were routinely grown with colchicine in the media, and drug was removed for 3 days prior to the start of all experiments. All cell lines were maintained in a humidified atmosphere with 5% CO_2_ at 37°C. All cultures were tested routinely and were mycoplasma-free.

Cells (1.25×10^6^ cells/10 cm dish) were plated and allowed to attach and grow for 3 days to reach 70–80% confluence. The cells were then trypsinised, washed in 10 mL PBS, centrifuged and the supernatant removed. The cell pellets were stored at −80°C prior to analysis. Total RNA was prepared using a RNeasy Mini Kit (Qiagen, UK) and the mirVANA miRNA Isolation Kit (Applied Biosystems) and expression was assessed as above.

### siRNA transfection and validation

The ovarian cancer cell line SKOV-3 was maintained in complete McCoy's media at 37°C in a humidified 5% CO_2_ atmosphere. Synthetic siRNAs against mRNA encoding MyD88 (siMyD88) and TLR4 (siTLR4), as well as negative control siRNA (siNeg) were obtained from Ambion. SKOV-3 (2.5×10^4^) cells were reverse transfected in 24-well plates with a final concentration of 1 nM siRNA using Lipofectamine RNAiMAX (Invitrogen). The transfected cells were incubated for 72 hrs before either harvesting for mRNA or protein analysis. Total RNA was isolated using the mirVana kit (Ambion). MyD88, TLR4 and B2M mRNA expression levels were evaluated by TaqMan RT-PCR. The data was analysed using the comparative ΔΔC_T_ method; where MyD88 and TLR4 mRNA expression was normalised to that of B2M and calibrated to that of untreated cells to establish the relative level of mRNA expression. Protein was isolated by cells lysis with RIPA buffer. The lysate samples (30 µg) were then resolved by SDS PAGE electrophoresis and transferred to a PVDF membrane. The membrane was then interrogated with antibodies specific for MyD88 (Cell Signalling), TLR4 (AbCam) or GAPDH (AbCam). GAPDH was used as a loading control.

### Drug treatment and cell viability assessment

72 hrs post transfection SKOV-3 cells were either left untreated, treated with DMSO (vehicle control) or 3.5 nM of paclitaxel (IC_25_). 48 hrs post treatment, cell viability was assessed by means of the CCK-8 assay (Sigma-Aldrich). Cell viability rate was calculated as the percentage of the absorption at 405 nm for each transfection condition, as follows: cell viability (% control)  =  (mean experimental absorbance/mean vehicle control cell absorbance) ×100.

### Embryonal Carcinoma Cell Culture

Pluripotent NTera2 and nullipotent 2102Ep embryonal carcinoma cells were maintained in the undifferentiated state in Dulbecco's Modified Eagle Media (DMEM) supplemented with 10% fetal calf serum (FCS), 5% L-Glutamine and 5% Penicillin-Streptomycin (Lonza, Basel, Switzerland), as per standard protocols. NTera2 cells were passaged via cell scrapping while 2102Ep cells were passaged via trypsinisation. Differentiation (NTera2) or differentiation-resistance mechanisms (2102Ep) were stimulated by the addition of 10 mM retinoic acid (RA) for 3 days. FFPE cell blocks were generated by rinsing and dispersing cell pellets in phosphate buffered saline (PBS) followed by fixation in 10% neutral buffered formalin (NBF) for 2 hours. Cells were then suspended in 1% agarose gel and processed and embedded according to routine laboratory procedures. RNA isolation, cDNA synthesis and TaqMan qPCR was carried out on embryonal carcinoma cell lines as previously described [29], using the TLR4, MyD88 and GAPDH assays listed above.

### Statistical analysis

SPSS for Windows, Rel. 16.0 2001. Chicago: SPSS Inc was used for statistical analysis of survival data. Kaplan-Meier survival curves were used; cases where the event had not occurred were censored. Log-rank was used for univariate analysis. Statistical analyses for qPCR used the Student's *t*-test, unpaired for normal distributions of at least three independent experiments. The criterion of statistical significance applied for all analyses was p<0.05. For gene silencing experiments data is expressed as means ± the standard deviation from at least three biological replicates. For all measurements as needed, a Student's t-test was used to assess the statistical significance of treated groups versus vehicle control groups. Student's t-test was performed using the GraphPad Prim version 5 software (GraphPad Software, CA, USA). A statistically significant difference was considered to be present at p<0.05.
