# Correction: The MyD88+ Phenotype Is an Adverse Prognostic Factor in Epithelial Ovarian Cancer

**DOI:** 10.1371/journal.pone.0108833

**Published:** 2014-09-17

**Authors:** The PLOS ONE Staff

The second to last author, Sharon O’Toole, should be noted as joint senior author of this work. The publisher apologizes for this error.
